# Differential tissue response to growth hormone in mice

**DOI:** 10.1002/2211-5463.12444

**Published:** 2018-05-23

**Authors:** Ryan Berry, Graham R. McGinnis, Ronadip R. Banerjee, Martin E. Young, Stuart J. Frank

**Affiliations:** ^1^ Department of Medicine Division of Endocrinology, Diabetes, and Metabolism University of Alabama at Birmingham AL USA; ^2^ Department of Medicine Division of Cardiovascular Disease University of Alabama at Birmingham AL USA; ^3^ Department of Cell, Developmental, and Integrative Biology University of Alabama at Birmingham AL USA; ^4^ Endocrinology Section Medical Service Veterans Affairs Medical Center Birmingham AL USA

**Keywords:** GH responsiveness, GH sensitivity, tissues

## Abstract

Growth hormone (GH) has been shown to act directly on multiple tissues throughout the body. Historically, it was believed that GH acted directly in the liver and only indirectly in other tissues via insulin‐like growth hormone 1 (IGF‐1). Despite extensive work to describe GH action in individual tissues, a comparative analysis of acute GH signaling in key metabolic tissues has not been performed. Herein, we address this knowledge gap. Acute tissue response to human recombinant GH was assessed in mice by measuring signaling via phospho‐STAT5 immunoblotting. STAT5 activation is an easily and reliably detected early marker of GH receptor engagement. We found differential tissue sensitivities; liver and kidney were equally GH‐sensitive and more sensitive than white adipose tissue, heart, and muscle (gastrocnemius). Gastrocnemius had the greatest maximal response compared to heart, liver, white adipose tissue, and whole kidney. Differences in maximum responsiveness were positively correlated with tissue STAT5 abundance, while differences in sensitivity were not explained by differences in GH receptor levels. Thus, GH sensitivity and responsiveness of distinct metabolic tissues differ and may impact physiology and disease.

**Abbreviations**
eWAT: epididymal white adipose tissue
gastroc: gastrocnemius
GH: growth hormone
IGF‐1: insulin‐like growth factor 1
JAK2: Janus kinase 2
MGF: mechano growth factor
PRLR: prolactin receptor
STAT5: signal transducer and activator of transcription 5

Growth hormone (GH), produced in the anterior pituitary, plays a major role in both longitudinal growth and metabolism 1, 2. Dysregulation in GH signaling, either increased in acromegaly and gigantism 3 or decreased in short stature or dwarfism, has profound consequences on growth and development 4, 5. GH also impacts life span; GH excess is associated with increased morbidity and premature mortality 6, while GH deficiency promotes longevity 7. GH binds cell surface receptors (GH receptor; GHR) on target cells, resulting in GHR‐associated Janus kinase 2 (JAK2) autophosphorylation and subsequent phosphorylation of GHR intracellular domain tyrosine residues 8, 9, 10, 11, 12, 13. Signal transducer and activator of transcription 5 (STAT5) docks at the phosphorylated GHR and is phosphorylated by JAK2. pSTAT5 dimers translocate to the nucleus to influence transcription of genes including insulin‐like growth factor (IGF)‐1 14, 15, 16; GH's metabolic and somatogenic effects are related to its influence on target cell gene expression.

Assessing acute GH effects in different key metabolic tissues may have once been considered an irrelevant question. Classically, GH was thought to exclusively target the liver, which would then produce IGF‐1 (aka somatomedin C) 17. IGF‐1 would subsequently act in an endocrine manner, modulating growth/metabolism in extrahepatic tissues. This is the somatomedin hypothesis of GH action 18. Later, D'Ercole *et al*. 19 showed that IGF‐1 is also produced locally by extrahepatic tissues in response to GH and that the level of IGF‐1 produced after GH administration differs between tissues. Further, Skottner *et al*. 20 demonstrated that administration of IGF‐1 did not affect longitudinal growth in hypophysectomized rats, except at very high concentrations, whereas GH administration induced significant growth. These pioneering studies suggested that IGF‐1 might be produced and act locally within target tissues, in contrast to the somatomedin hypothesis. Consistent with these observations, liver‐specific IGF‐1 knockout mice grow and develop normally, despite diminished circulating IGF‐1 21, 22, 23. As such, a revised hypothesis suggests that circulating (hepatic‐derived) IGF‐1 is responsible for negatively regulating GH secretion, whereas local (extrahepatic) IGF‐1 plays a primary role in longitudinal growth 24.

Despite interest in extrahepatic actions of GH and IGF‐1, little information is available that compares GH signaling among organs in intact animals. Because of the distinct roles of GH signaling in the liver compared to other metabolic tissues, we hypothesized that GH sensitivity and responsiveness would differ in hepatic versus extrahepatic tissues. Herein, we compare acute *in vivo* sensitivity and MAX responsiveness to exogenously administered GH in mice among liver, heart, kidney, skeletal muscle (gastrocnemius; gastroc), and epididymal white adipose tissue (eWAT). Our results indicate substantial differences between tissues that may be important for understanding tissue‐specific metabolic and growth‐promoting effects of GH.

## Materials and methods

Unless otherwise stated, reagents were obtained from Sigma (St. Louis, MO).

### Animals

All animal husbandry and experimental protocols were carried out according to the Guide for the Care and Use of Laboratory Animals [1996 (7th ed.), Washington, DC: National Research Council, National Academies Press] and in compliance with the local IACUC standards. At 15 weeks of age (± 3 days), male C57B6J mice (Jackson Laboratories; Cat. # 000664) were individually housed in standard conditions under a 12‐h : 12‐h light:dark cycle and had *ad libitum* access to standard rodent chow and water. After acclimatization to single housing, mice were placed in wire‐bottom cages without food at the beginning of the light cycle. Growth hormone challenge was performed in 6‐h fasted mice in a manner that is essentially identical to that described previously 25. Briefly, either saline (control) or human recombinant GH (2, 4, 8, 12.5, 20, 50, 80, 120, 200 ng/g_bw_; gift from Eli Lilly Co, Indianapolis, IN) was injected (i.v.) in anesthetized mice; 5 min thereafter, heart, liver, kidney, eWAT, and gastroc were rapidly excised in that order and flash‐frozen in liquid nitrogen prior to biochemical analysis. Total time of tissue extraction for each animal was 3–4 min. The duration of GH exposure was selected so as to capture only acute (and not secondary) effects of GH stimulation and thus most cleanly address the question of GH sensitivity.

Liver samples for PRLR mRNA positive control were harvested from female C56Bl6/J mice that were *ad libitum* fed and age‐matched, age 2–3 months. Pregnant samples were harvested at gestational day 16.5.

This study protocol was approved by the University of Alabama at Birmingham Institutional Animal Care and Use Committee.

### Immunoblotting

Protein lysates were prepared from tissues crushed to powder under liquid nitrogen (~ 20 mg) using 300 μL of tissue lysis buffer (50 mm Tris 7.3, 150 mm NaCl, 1 mm EDTA pH 8.1, 1.5 mm MgCl_2_, 10% glycerol, 1% Triton X‐100, 10 mm Na_4_P_2_O_7_, 100 mm NaF, 1 mm Na_3_VO_4_, 1 mm phenylmethanesulfonyl fluoride, 5 μg·mL^−1^ aprotinin, and 5 μg·mL^−1^ leupeptin). Lysates were resolved under reducing conditions by SDS/PAGE and transferred to nitrocellulose membranes (Amersham Biosciences), followed by blocking with 2% BSA. Membranes were immunoblotted (Table 1) with anti‐phospho‐STAT5 antibody (Y694; Cell Signaling; 9351L) (1 : 1000), which reacts with both phosphorylated Y694 in STAT5A and Y699 in STAT5B; anti‐STAT5 antibody (Santa Cruz Biotechnology; sc‐835) (1 : 1000); anti‐GHR (polyclonal anti‐GHR_cytAL‐47_; against the intracellular domain of GHR) 26 (1 : 1000); anti‐PRLR (anti‐PRLR_cytAL‐84_; against the human PRLR ICD) 27 (1 : 1000); anti‐PRL‐R (H‐300) (Santa Cruz Biotechnology; sc‐20992); and anti‐JAK2 (anti‐JAK2_AL‐33_) 28 (1 : 1000). Densitometry was performed using UVP Software 8.0.

**Table 1:** Antibody table

Antigen sequence (if known)	Name of antibody	Manufacturer, catalog #, and/or name of individual providing the antibody	Species raised in; monoclonal or polyclonal	Dilution used	RRID
Y694, mouse, rat, bovine, human	Anti‐pSTAT5	Cell Signaling, 9351L	Rabbit; polyclonal	1 : 1000	AB_331594
Stat5 (C‐17) human, mouse, rat	STAT5 (C‐17)	Santa Cruz, Cat. # sc‐836	Rabbit; polyclonal	1 : 1000	AB_632446
Residues 271‐620	Anti‐GHR_cytAL‐47_	Stuart J. Frank	Rabbit; polyclonal	1 : 1000	AB_2713931
PRLR intracellular domain	Anti‐PRLR_cytAL‐48_	Stuart J. Frank	Rabbit; polyclonal	1 : 1000	AB_2665406
Residues 323‐622	PRL‐R (H‐300)	Santa Cruz, Cat. # sc‐20992	Rabbit; polyclonal	1 : 1000	AB_2237692
Residues 746‐1129	Anti‐JAK2_AL‐33_	Stuart J. Frank	Rabbit; polyclonal	1 : 1000	AB_2665398

### Curve fitting and statistical analysis

Dose–response curve data were fit to the sigmoidal dose–response curve (with variable slope): [Y = BOTTOM + (TOP‐BOTTOM)/(1 + 10^((LogEC_50_‐X)*HillSlope))]; Y = response; X = log[dose]; HillSlope = slope of linear section of the dose–response curve; TOP = point in the dose–response curve at which an increase in ‘X’ yields little to no increase in ‘Y’; and EC_50_ = the effective concentration (or dose) at which 50% of the MAX response is achieved 29. This analysis was performed using GraphPad Prism version 4.00 for Windows (GraphPad Software, San Diego, California, USA, http://www.graphpad.com). Sensitivity was defined by the value EC_50_. Responsiveness was defined by the TOP value, herein referred to as the MAX response. During the constrained fit, the TOP and BOTTOM parameters were fixed at 100 and 0, respectively. Tissue‐specific differences in protein abundances were assessed via one‐way ANOVA using SPSS followed by post hoc analysis via Tukey's test. Regression analysis to assess the correlation between STAT5 abundance and MAX response was performed using Excel.

### Gene analysis

mRNA was isolated from mouse tissues using either a QIAGEN RNeasy Mini Kit (Cat. No. 74104) or TRIzol RNA isolation reagent according to the manufacturer's recommended protocol for RNA isolation. Reverse transcription was performed using the High‐Capacity cDNA RT Kit (Cat. No. 4368814) from Thermo Fisher. qPCR measurements were carried out using the mPRLR TaqMan Gene Exp. Assay (Assay ID Mm04336676_m1, Cat. No. 4351372) from Thermo Fisher.

### Data display

Due to differences in normalizing proteins across tissues, densitometry data are normalized to total protein loaded on the gel except in Fig. 2 where pSTAT5 is normalized to STAT5 as a loading control; tissue differences in STAT5 abundance do not influence sensitivity. To reduce positional bias during the immunoblot transfer procedure, samples were loaded on gels in randomized order; where possible, *n* = 1 for each GH dose was included on each gel. Densitometry was performed on nonmanipulated blots. For clarity, representative blots presented were constructed as follows: A single gel was chosen for each tissue, after which lanes were rearranged such that GH doses were displayed in ascending order.

## Results

### Murine peripheral tissues display differential sensitivity and MAX response to GH

**Figure 1:**
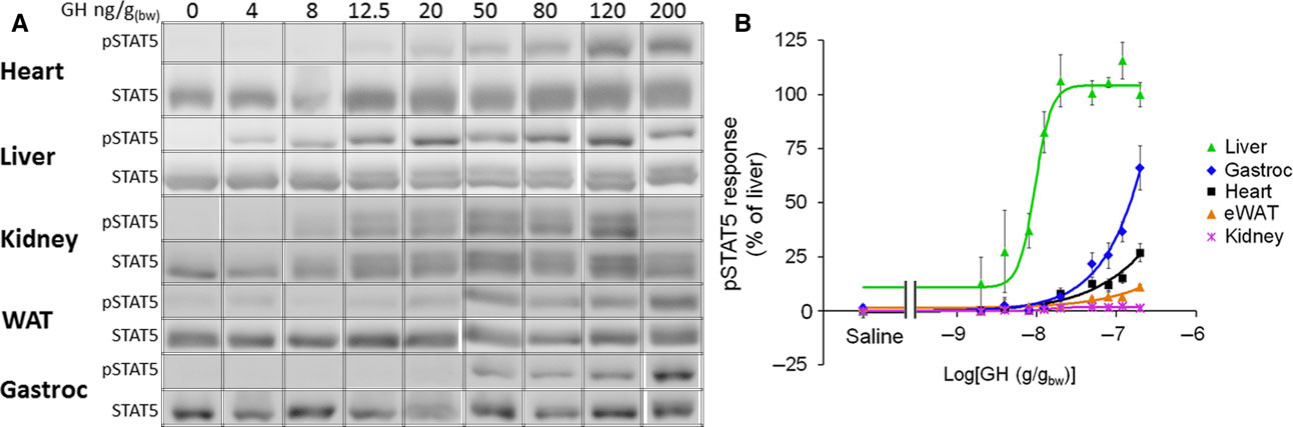
Tissue GH sensitivity and responsiveness (free fit). (A) Representative blots for dose‐dependent GH‐induced STAT5 phosphorylation in liver, kidney, eWAT, heart, and gastrocnemius muscle. The exposure of the blots was adjusted to be able to visualize dose dependencies of the different tissues, and as such, the intensities of bands may not be compared between tissues. (B) Dose–response data from liver, kidney, eWAT, heart, and gastrocnemius fit to the Hill equation without fit constraints (mean ± SEM; *n* = 3–10).

**Figure 2:**
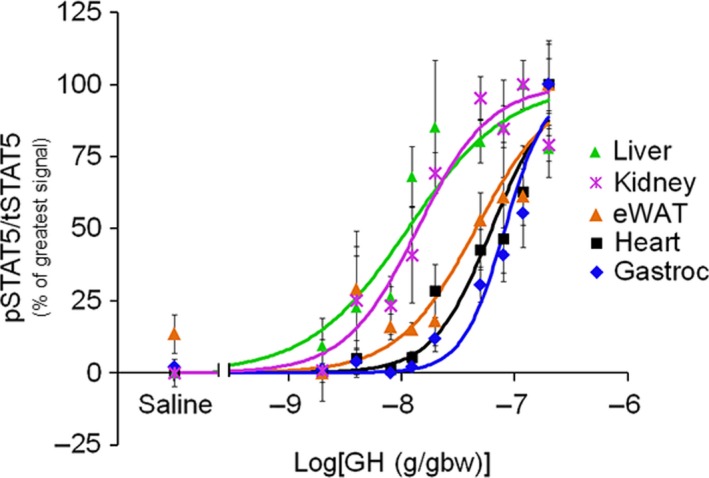
Tissue GH sensitivity (constrained fit). Dose–response data normalized such that the highest value within each tissue is 100 and lowest is 0, and fit to the Hill equation with the constraints: TOP = 100 and BOTTOM = 0.

Although they varied greatly in responsiveness, with kidney being the least maximally responsive, all tissues examined displayed dose‐dependent GH effects on STAT5 phosphorylation (Figs 1A and 2). Calculation of EC_50_ values (see the Methods section for details) revealed tissue‐specific differences in GH sensitivity (Fig. 1B, Table 2). EC_50_ values for liver and kidney did not differ significantly, although both were substantially lower (i.e., greater sensitivity) than eWAT, heart, and gastrocnemius. Differences were likewise observed in tissue responsiveness (Fig. 1B, Table 2). Gastrocnemius had the greatest (extrapolated) MAX GH response, followed by heart, liver, eWAT, and kidney (Fig. 1B, Table 2). There was large variability (i.e., confidence intervals) in the EC_50_ and MAX values for gastrocnemius, heart, and eWAT because the predicted MAX value was not defined by experimental data points (as predicted GH doses required for MAX response were too high). Therefore, as a secondary analysis we normalized the data for each curve such that the highest experimental data point was 100 while the lowest was 0, and fit the data to the sigmoidal dose–response curve using the constraints TOP = 100 and BOTTOM = 0 (Fig. 2). This analysis yielded similar EC_50_ calculations for liver and kidney, as well as similar R^2^ values for all curve fits. Furthermore, it confirmed, statistically, that liver and kidney have the same EC_50_ and that they are significantly more sensitive than eWAT, heart, and gastrocnemius (Fig. 2, Table 2).

**Table 2:** Curve fit parameters: from fitting dose–response data from Fig. 1B to the Hill equation without constraints (free fit), and from Fig. 2 using fit constraints (constrained fit)

	Liver	Kidney	eWAT	Heart	Gastroc
Free fit					
EC_50_ (ng/g_bw_)	10	14	1248	4901	1642
MAX response (A.U.)	104.3	1.8	53.6	296.1	615.2
R^2^	0.8622	0.65	0.6343	0.6928	0.7379
Constrained fit					
EC_50_ (ng/g_bw_)	11	14	46	63	82
EC_50_ (ng/g_bw_) 95% CI	7.7–16.9	9.2–20.9	30.9–67.1	47.0–83.9	65.0–103.5
R^2^	0.7344	0.6744	0.6288	0.722	0.7241

### Differential abundance of GH signaling proteins among tissues

**Figure 3:**
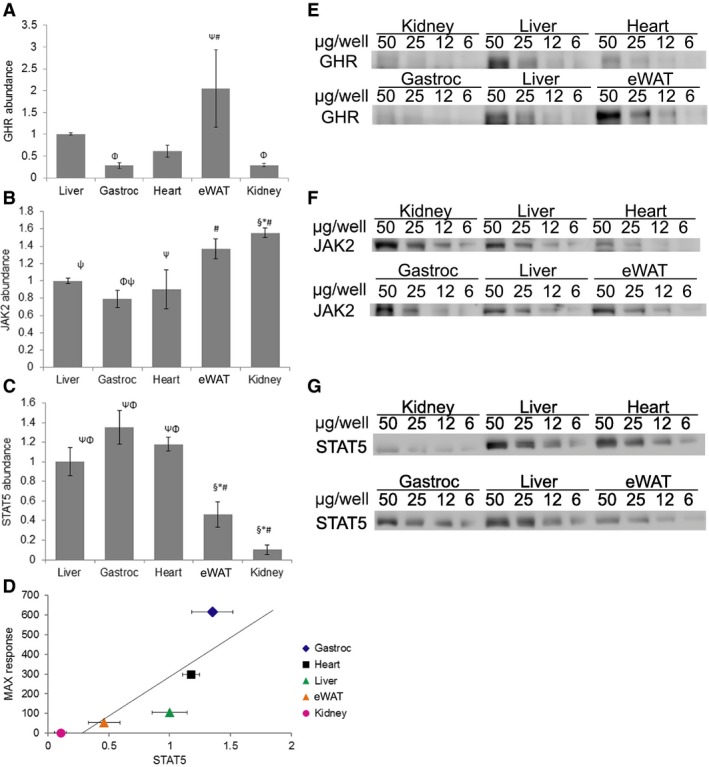
GH signaling components. Densitometry analysis and representative blots of tissue‐specific comparison of protein abundance for (A,E) GHR, (B,F) JAK2, and (C,G) STAT5 (relative to liver) (mean ± SEM; *n* = 5). Significance: All symbols represent *P* < 0.05 compared to: *, liver; #, gastrocnemius; §, heart; Φ, eWAT; and Ψ, kidney. (D) Linear regression analysis displaying the correlation between tissue‐specific MAX response and STAT5 (mean; *n* = 5) abundance (correlation coefficient: + 0.83; *P* = 0.082).

**Figure 4:**
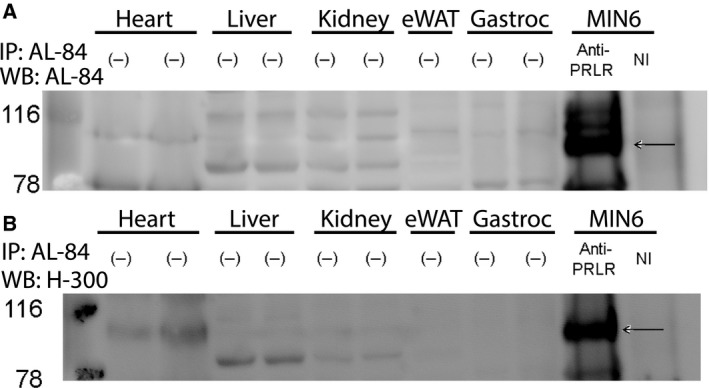
PRLR immunoblot of tissue lysates from various mouse tissues and in MIN6 mouse insulinoma cells (positive control). The black arrows denote the PRLR in the IP control. (−) denotes that no immunoprecipitation was performed. NI denotes nonimmune serum. Immunoprecipitation was performed with anti‐PRLR_cytAL‐84_, and resolved eluates were immunoblotted sequentially with (A) anti‐PRLR_cytAL‐84_ and (B) anti‐PRL‐R (H‐300).

**Figure 5:**
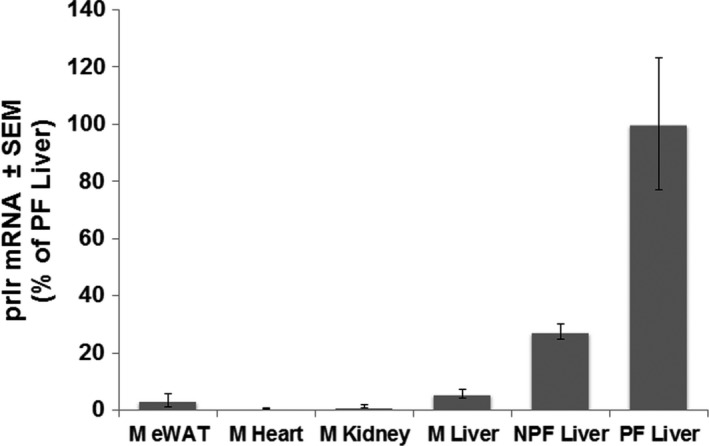
Comparison of PRLR mRNA expression between the male mouse tissues and with livers from pregnant and nonpregnant female mice. M, male; NPF, nonpregnant virgin female; PF, pregnant female. Data represented as mean ± SEM; *n* = 3‐5 for each condition.

The factors that influence tissue sensitivity to a hormone often reside at the level of the receptor. Accordingly, we assessed GHR abundance by immunoblotting with an antibody against the GHR intracellular domain, which revealed highest abundance in eWAT (2.05 A.U.), followed by liver (1.00 A.U.), heart (0.61 A.U.), kidney (0.29 A.U.), and gastrocnemius (0.28 A.U.) (Fig. 3A,E). This study utilized human GH, which can also induce STAT5 phosphorylation via the prolactin receptor (PRLR) 30, 31, 32. We compared PRLR‐expressing MIN6 cells to the relevant mouse tissues by immunoblotting with two distinct anti‐PRLR sera (anti‐PRLR_cytAL‐84_ and anti‐PRL‐R (H‐300); Fig. 4A,B, respectively). No bands in common were detected by these sera in the mouse tissues, but a common PRLR band was detected by both in the MIN6 positive control. Analysis of *prlr* mRNA levels validated the conclusion that little or no expression was detected in the mouse tissues tested (Fig. 5). Thus, analyses of GHR and PRLR abundance did not readily explain observed tissue‐specific differences in GH sensitivity (although relatively high GHR expression in the liver may contribute to elevated GH sensitivity in this tissue).

In contrast to hormone sensitivity, the responsiveness of a tissue to a hormone is influenced by factors downstream of the receptor, including abundance of downstream signaling molecules. To this end, we assessed JAK2 and STAT5 abundance by immunoblotting. Relatively modest differences were observed in JAK2 abundance, with lowest levels in gastrocnemius and highest levels in kidney (Fig. 3B,F). STAT5 abundance did not differ between liver, gastrocnemius, and heart, but was significantly lower in eWAT and kidney (Fig. 3C,G). Regression analysis revealed a correlation (correlation coefficient: + 0.8296) between the STAT5 abundance in a tissue and its MAX response (*P* = 0.082) (Fig. 3D).

## Discussion

The purpose of the current study was to define tissue‐specific differences in GH sensitivity and MAX responsiveness to GH. Here, we report that the order of GH sensitivity was liver = kidney > eWAT = heart = gastrocnemius, while the order of GH MAX responsiveness was gastrocnemius > heart > liver > eWAT > kidney and roughly correlated with STAT5 protein abundance. Such observations lead to questions with regard to physiologic significance. While the MAX response predicted from the free curve fitting in gastrocnemius and heart was much greater than in the other tissues, the levels of GH required to attain that MAX stimulation are far beyond physiologic levels. However, despite the error in these predicted values being large, the fact that there was a near‐significant correlation between STAT5 and MAX response supports the idea that these values are good estimates. The liver and kidney exhibit the highest level of GH sensitivity (relative to other tissues investigated), and the other three tissues were indistinguishable statistically. That this general relationship holds true regardless of whether constraints were used supports the idea that the liver and kidney respond to GH at much lower concentrations than eWAT, heart, and gastrocnemius.

Growth hormone plays a number of important roles in the liver, including generation of circulating IGF‐1 (which acts in a negative feedback manner on GH secretion) and hepatic metabolism. In the latter case, GH effects generally oppose those of insulin; specifically, these effects suppress glycolysis in favor of fatty acid oxidation and promote glycogenolysis and in prolonged fasting conditions promote gluconeogenesis 1, 33, 34, 35, 36. Thus, increased GH secretion during sleep likely plays an important role in maintenance of blood glucose levels via multiple mechanisms. Interestingly, the kidney is also a gluconeogenic tissue, contributing up to 50% of endogenous glucose production in the starved state 37. GH signaling in the kidney is also important for normal sodium and water retention; GH deficiency leads to renal insufficiency, while excess leads to hypertension, renal hypertrophy, and failure 37. Thus, our observation that the kidney is relatively GH‐sensitive (similar to the liver) is consistent with essential GH actions in this tissue. GH signaling is also important in eWAT, as this endocrine factor shifts metabolism from glucose utilization toward lipolysis and fatty acid oxidation, thereby minimizing reliance on muscle protein catabolism during periods of fasting (such as the sleep period) 1, 38, 39, 40. In contrast, GH signaling in the adult heart must be closely regulated, thus preventing excessive growth (e.g., in acromegaly) and subsequent contractile dysfunction 41. Similar to the heart, GH signaling in skeletal muscle mainly influences muscle size, but not contractile force 38, 40, 42. Our observation that skeletal muscle has decreased responsiveness to circulating GH may be explained by the existence of mechano growth factor (MGF), an alternative splice variant of the *igf‐1* gene. MGF expression is increased in response to muscle stretch and exercise 43. Even hypophysectomized mice retain the ability to upregulate MGF in response to exercise 43, 44. The low GH sensitivity of gastrocnemius muscle may suggest that skeletal muscle growth in an adult mouse, in response to exercise, for example, may be through GH‐independent mechanisms.

Subsequent interrogation of known GH signaling components provided potential mechanistic insights with regard to tissue‐specific differences in GH responsiveness/sensitivity. For example, STAT5 levels were correlated with MAX response in a given tissue. Additionally, GHR levels were relatively high in liver, consistent with high GH sensitivity. Our findings are consistent with those of Walker *et al*. 45, who reported that GHR mRNA in the rat kidney was roughly 33% that of liver. Additional studies are required to elucidate fully the mechanisms mediating tissue‐specific differences in GH sensitivity/responsiveness.

The current study focused on a particular acute signaling response of various tissues to exogenously administered GH (namely STAT5 phosphorylation). This approach has benefits and drawbacks. Although we did not assess the long‐term response to endogenous GH pulses, this approach allowed us to directly compare acute responses to GH in multiple tissues simultaneously. As STAT5 is a critical mediator of acute GH action, we were able to observe direct GH effects, rather than compensatory effects over longer periods. Nonetheless, we acknowledge that our studies do not discriminate between the STAT5A and STAT5B isoforms of STAT5. As different tissues may express varying ratios of these isoforms, our conclusions concerning maximum responsiveness based on STAT5 abundance should be interpreted with caution.

As noted above, GH stimulates glycogenolysis in liver and kidney during fasting 35, 36. The mice in this study were fasted for 6 h prior to GH treatment. Therefore, it is possible that we would have observed a different relationship among tissues of GH sensitivity in mice if food had not been withdrawn in the 6 h leading up to GH treatment. However, the period of fasting corresponded to the first 6 h of the rest phase, during which food consumption is generally reduced (relative fasting), compared to the active period 46. Thus, the relative physiologic effects of the strict fast are likely limited. We are mindful, however, that GH sensitivity and MAX response were only assessed at one time of day in our study. Because the circadian clock may control both secretion and sensitivity to hormones 47, it is possible that relative tissue sensitivity to GH may vary depending on the time of day.

In summary, the current study reveals a correlation between STAT5 abundance and the MAX GH response in these tissues, while GH sensitivity is not correlated with GHR. Thus, an important determinant of MAX GH response appears to be STAT5 abundance, while the determinants of *in vivo* GH sensitivity are more complex. We speculate that in pathological states, GH action may be influenced by alterations in GH sensitivity and/or responsiveness, not solely by changes in circulating GH levels. Our data from wild‐type mice will serve as a template for analyzing such changes in disease states.

## Author contributions

RDB, MEY, and SJF conceived and designed the project, analyzed and interpreted the data, and wrote the manuscript. RDB, RRB, and GRM acquired the data.
